# Bidirectional Mast Cell–Eosinophil Interactions in Inflammatory Disorders and Cancer

**DOI:** 10.3389/fmed.2017.00103

**Published:** 2017-07-24

**Authors:** Maria Rosaria Galdiero, Gilda Varricchi, Mansour Seaf, Giancarlo Marone, Francesca Levi-Schaffer, Gianni Marone

**Affiliations:** ^1^Department of Translational Medical Sciences (DiSMeT), Center for Basic and Clinical Immunology Research (CISI), University of Naples Federico II, Naples, Italy; ^2^Pharmacology and Experimental Therapeutics Unit, Faculty of Medicine, School of Pharmacy, Institute for Drug Research, The Hebrew University of Jerusalem, Jerusalem, Israel; ^3^Department of Public Health, University of Naples Federico II, Monaldi Hospital Pharmacy, Naples, Italy; ^4^Institute of Experimental Endocrinology and Oncology “Gaetano Salvatore” (IEOS), National Research Council (CNR), Naples, Italy

**Keywords:** allergy, asthma, cancer, eosinophils, inflammation, mast cells, mastocytosis

## Abstract

Human mast cells (MCs) and eosinophils were first described and named by Paul Ehrlich. These cells have distinct myeloid progenitors and differ morphologically, ultrastructurally, immunologically, biochemically, and pharmacologically. However, MCs and eosinophils play a pivotal role in several allergic disorders. In addition, these cells are involved in autoimmune disorders, cardiovascular diseases, and cancer. MCs are distributed throughout all normal human tissues, whereas eosinophils are present only in gastrointestinal tract, secondary lymphoid tissues, and adipose tissue, thymus, mammary gland, and uterus. However, in allergic disorders, MCs and eosinophils can form the “allergic effector unit.” Moreover, in several tumors, MCs and eosinophils can be found in close proximity. Therefore, it is likely that MCs have the capacity to modulate eosinophil functions and *vice versa*. For example, interleukin 5, stem cell factor, histamine, platelet-activating factor (PAF), prostaglandin D_2_ (PGD_2_), cysteinyl leukotrienes, and vascular endothelial growth factors (VEGFs), produced by activated MCs, can modulate eosinophil functions through the engagement of specific receptors. In contrast, eosinophil cationic proteins such as eosinophil cationic protein and major basic protein (MBP), nerve growth factor, and VEGFs released by activated eosinophils can modulate MC functions. These bidirectional interactions between MCs and eosinophils might be relevant not only in allergic diseases but also in several inflammatory and neoplastic disorders.

## Introduction

Mast cells (MCs) and eosinophils are important cells of the immune system with critical roles in allergic (1–3) and autoimmune disorders (4–7), cardiovascular diseases (8–15), and cancer (16–19). Human MCs and eosinophils were first described and named in 1878 and 1879, respectively, by Paul Ehrlich who discovered their property to be stained by specific dyes (20–22). Mature MCs and eosinophils differ morphologically, ultrastructurally, immunologically, biochemically, and pharmacologically (23, 24). Moreover, they synthesize a plethora of distinct mediators and display a constellation of different surface receptors (24, 25).

The recent assessment of the transcriptional profiles of MCs and eosinophils revealed the MC heterogeneity across different tissues and their different gene expression program compared to eosinophils (26). The latter findings are consistent with the identification of a distinct myeloid progenitor expressing the gene encoding the transcription factor GATA-1, which generates separately eosinophils and MCs (27). Human MCs derive from CD34^+^, CD117^+^ pluripotent hematopoietic stem cells in the bone marrow (28). MC progenitors enter the circulation and complete their maturation in different tissues such as skin, bronchi, tonsils, nasal and intestinal mucosa, conjunctiva, lymph nodes, and breast parenchyma (29). The main differentiation, maturation, survival, priming, and chemotactic factor for human MCs is stem cell factor (SCF), which acts by binding the tyrosine kinase receptor Kit (CD117) (30). CD34^+^ IL-5Rα^+^ eosinophil lineage-committed progenitors give rise to mature cells in the bone marrow under the control of critical transcription factors such as GATA-1, PU-1, and C/EBP (31). Eosinophil maturation in the bone marrow is driven by interleukin 5 (IL-5), IL-3, and granulocyte-macrophage colony-stimulating factor (GM-CSF) that share the common receptor β subunit (βc) (3, 32). Recent evidence indicates that IL-33 precedes IL-5 in regulating eosinophil commitment and is required for eosinophil homeostasis (33). Under the effect of chemotactic stimuli, together with IL-5, eosinophils migrate to the gastrointestinal tract, secondary lymphoid tissues, and adipose tissue, thymus, mammary gland, and uterus, where they reside under homeostatic conditions (see Marichal et al. in this issue) (34–36). In response to inflammatory stimuli (e.g., eotaxins), peripheral blood eosinophils migrate into inflamed tissues, where their survival is presumably prolonged (37, 38).

## Bidirectional MC–Eosinophil Interactions

Although human MCs are distributed throughout, nearly all normal tissues (39, 40) their density is increased at sites of allergic reactions (1), autoimmune disorders (4, 5), and at the edge of several solid (41–55) and hematologic tumors (56–64). In several allergic disorders (e.g., bronchial asthma, allergic rhinitis, chronic urticaria, and eosinophilic esophagitis), MCs and eosinophils can be found in close proximity forming the “allergic effector unit” (AEU) (65). In addition, there is *in vitro* evidence that the physical interaction between MCs and eosinophils induces a hyperactivation state and release of soluble mediators (65–67). Therefore, MCs likely have the capacity to modulate eosinophil functions and *vice versa*. We discuss examples of such two-way interactions below.

### MC Mediators

Histamine, released immunologically and non-immunologically from MCs, induces eosinophil chemotaxis through the engagement of the histamine 4 receptor (H_4_R) (68, 69). Similar to eosinophils, histamine-induced chemotaxis can be also observed in MCs (69).

Adenosine, an endogenous nucleoside released by activated MCs (70), acts in an autocrine and paracrine fashion *via* binding to four G protein-coupled receptors: the A_1_, A_2a_, A_2b_, and A_3_ receptors (71) and is involved in airway hyperresponsiveness in asthma (72). Adenosine and its stable analogs potentiate mediator release from human lung MCs (HLMCs) (73, 74) through the activation of adenosine receptors (75) and modulate eosinophil functions (76, 77). MC tryptase can stimulate eosinophil activation and degranulation by cleavage of protease-activated receptor 2 (78).

### Eosinophil Mediators

On the other side, eosinophil granule proteins such as MBP and eosinophil cationic protein (ECP) act as complete secretagogues on MCs isolated from human heart (HHMC) (8, 9). ECP, and to a lesser extent MBP, induces the release of histamine and tryptase and the *de novo* synthesis of PGD_2_ from HHMC. This observation highlights a mechanism by which infiltrating eosinophils can cause myocardial damage in patients with eosinophilia (3, 79–84). ECP and MBP do not induce histamine release from isolated HLMCs (8, 9). Interestingly, Piliponsky et al. reported that HLMCs became responsive to MBP only in coculture with human lung fibroblasts (85). Recently, the Mas-related gene X2 (MRGPRX2) has been identified as a receptor for several basic peptides on human and rodent MCs (26, 86), and indeed ECP and MBP activate human MCs through the interaction of the MRGPRX2 receptor expressed on their surface (87). Eosinophil MBP-1 activates MCs through the interaction with integrin-β1 (88).

### MC and Eosinophil Mediators

Stem cell factor (SCF) is a potent activator of human MCs (89, 90) and induces the release of eosinophil peroxidase (EPO) and cysteinyl leukotriene C_4_ (LTC_4_) from eosinophils (91). SCF, produced by both human MCs (90) and eosinophils (92), acts on Kit receptor (CD117) on MCs (30) and eosinophils (93).

Osteopontin (OPN) is a multifunctional glycoprotein implicated in allergic disorders and cancer. OPN can be released by IL-5-activated human eosinophils and induces their migration (94). OPN is also produced by MCs and modulates their IgE-mediated degranulation and migration (95).

Interleukin-5, produced by human MCs, activates the IL-5R, highly expressed on the surface of human eosinophils, basophils, and MCs (96). In addition to MCs, Th2 cells, group 2 innate lymphoid cells (ILC2), invariant NK T cells, and eosinophils themselves are major cellular sources of IL-5 (97). GM-CSF released by activated human MCs (98), and eosinophils binds its receptor expressed by both cell types (99). The cysteinyl leukotrienes (CysLTs LTC_4_ and LTD_4_), produced by activated MCs (18, 100), stimulate the proliferation of eosinophil progenitors in the presence of IL-5 and GM-CSF (101). In addition, CysLTs acting through CysLTR1/2 induce the release of IL-4 from human eosinophils (102). PGD_2_ is the major cyclooxygenase metabolite released by activated MCs (8) and a minor product of eosinophils (103). PGD_2_ is involved in asthma and allergic rhinitis (104, 105), mastocytosis, rheumatoid arthritis, and cardiac dysfunction (6, 106). PGD_2_ induces eosinophil and MC chemotaxis in a paracrine and autocrine fashion *via* binding to CRTH2 receptor on these cells (107, 108). Platelet-activating factor (PAF), synthesized by human MCs and eosinophils (109, 110), is involved in asthma (111) and exerts multiple effects on eosinophils (112, 113).

Nerve growth factor (NGF), produced by both MCs (114, 115) and eosinophils (116, 117), is increased in patients with asthma (118). NGF enhances MC survival (119) through the activation of TrkA receptor (115). NGF is preformed in and activates human eosinophils (116).

Human MCs produce several proangiogenic (VEGF-A, VEGF-B, and FGF-2) (120–125) and lymphangiogenic factors (VEGF-C and VEGF-D) (100, 124). Human eosinophils induce angiogenesis (126) through the production of VEGF-A (127, 128), MBP (129), and OPN (94). Interestingly, VEGF-A, produced by both MCs and eosinophils, is also chemotactic for MCs through the engagement of VEGFR-1/2 present on their surface (124).

**Figure 1:**
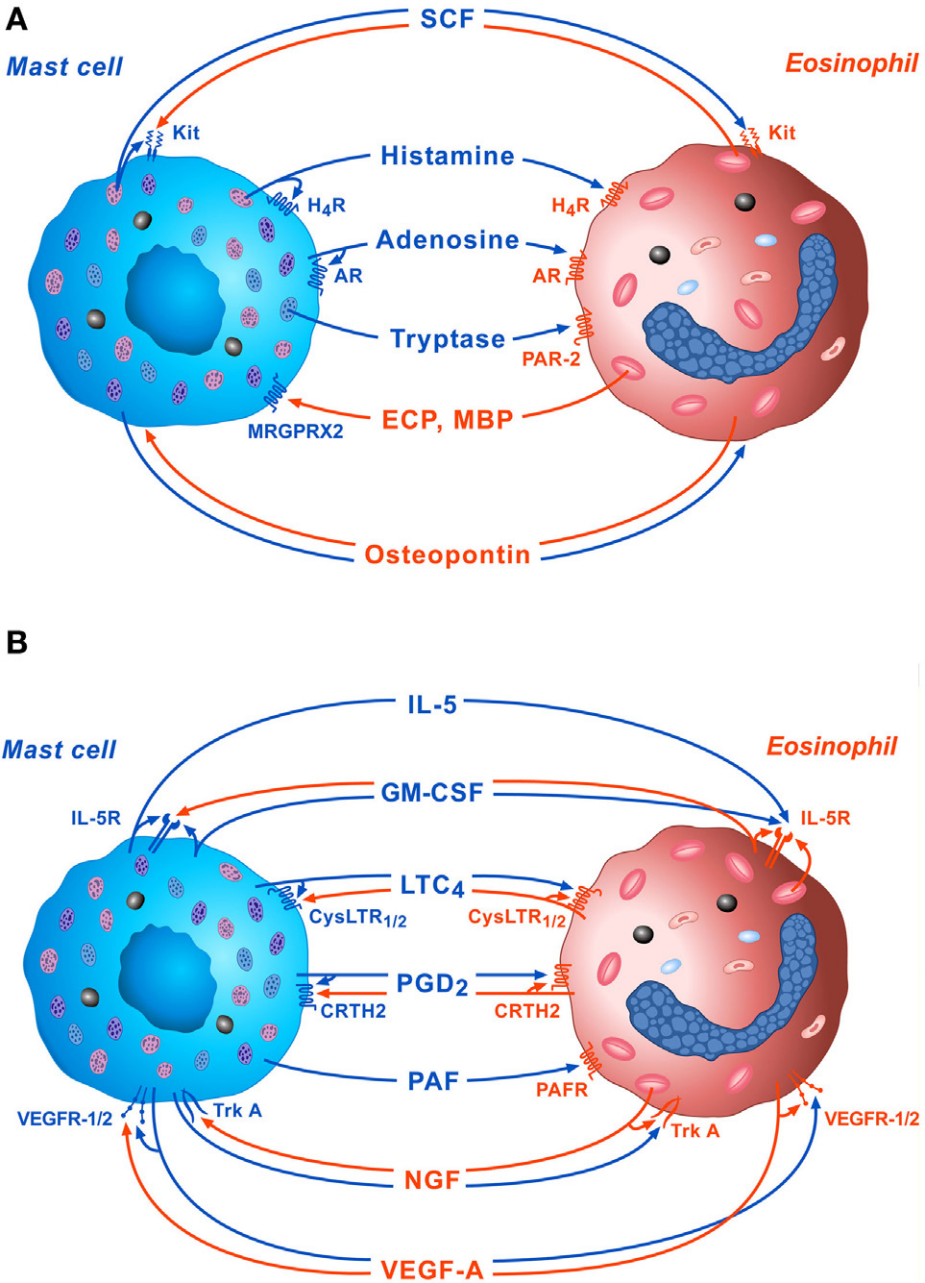
Schematic representation of some of the bidirectional interactions between MCs and eosinophils. **(A)** Several preformed mediators such as stem cell factor (SCF), histamine, adenosine, and tryptase, released by activated MCs can exert paracrine and/or autocrine functions through the engagement of Kit, H_4_R, adenosine receptors, and protease-activated receptor 2 (PAR-2), respectively. On the other side, cationic proteins [eosinophil cationic protein (ECP) and MBP], released by activated eosinophils modulate mast cell functions through the activation of MRGPRX2 on their surface. Osteopontin released by both activated eosinophils and MCs exert paracrine and autocrine effects. **(B)** Several *de novo* synthesized mediators such as IL-5, granulocyte-macrophage colony-stimulating factor (GM-CSF), LTC_4_, PGD_2_, platelet-activating factor (PFA), nerve growth factor (NGF), and VEGF-A, released by activated MCs, can modulate eosinophil functions *via* the activation of IL-5R, CysLTR_1/2_, CRTH2, platelet-activating factor receptor (PAFR), TrkA, and VEGF-R1/2, respectively, on their surface. IL-5, GM-CSF, LTC_4_, PGD_2_, NGF, and VEGF-A can also exert paracrine and/or autocrine effects.

The bidirectional interactions between MCs and eosinophils mediated by soluble mediators and the autocrine modulation of these cells are schematically illustrated in Figures 1A,B.

## Disorders in Which MCs and Eosinophils are Present and Likely Drive Disease Pathogenesis

### Asthma

Asthma is a chronic inflammatory disorder of the airways in which cells of the innate and adaptive immune system act together with epithelial cells to cause bronchial hyperreactivity, mucus overproduction, airway wall remodeling, angiogenesis, and airway narrowing (123, 130, 131). MCs and their mediators display important roles in the pathogenesis of asthma (2, 39). Indeed, MC-derived histamine, proteases, chemotactic factors, cytokines, and metabolites of arachidonic acid act on vasculature, smooth muscle, connective tissue, goblet cells, and inflammatory cells in the airway inducing acute bronchoconstriction (1). MCs synthesize and release a vast array of pro-inflammatory chemokines and cytokines and recruit other immune cells, such as eosinophils, activated macrophages, and lymphocytes. Therefore, MCs are involved both in the early and the late phases of allergic responses in sensitized individuals (132). Compelling evidence suggests that in asthma MCs are constantly activated resulting in enhanced mediator release and the establishment of chronic airway inflammation. Moreover, MCs reside close to key structures of the bronchial wall, such as airway smooth muscle (ASM) epithelium and submucosal glands, contributing to ASM hypertrophy and other remodeling features (133).

Numerous stages of the MC life cycle have the potential for therapeutic intervention in allergic disorders (134). Targeting the progenitor recruitment offers an upstream checkpoint that could be used to limit tissue MC activity. However, since the mechanisms regulating MC progenitor recruitment to the human lung are not fully understood, no potential therapeutic targets at this level of MC biology have been defined so far. Once within tissue, MC survival, growth, differentiation, and maturation are driven by the local cytokine melieu, with a pivotal role played by SCF and its receptor Kit, which retains protein tyrosine kinase (TK) activity. MC eradication *via* TK inhibitors may also be a means to treat MC-driven diseases such as asthma. Indeed, the TK inhibitor imatinib decreased airway hyperresponsiveness, MC counts, and tryptase release in patients with severe asthma (135). In addition, masitinib, an inhibitor of Kit and the platelet-derived growth factor receptor, showed some benefit in a small phase II trial over 16 weeks in severe glucocorticoid-dependent asthma (136).

High-affinity receptor for the Fc region of IgE (FcεRI) is expressed on MCs and basophils as a tetrameric complex of three chains with the stoichiometry αβγ_2_. FcεRI is also expressed in either a trimeric form, αγ_2_, or the tetramer, on a range of other cell types [e.g., various antigen-presenting cells (APCs), dendritic cells, Langerhans cells, macrophages, eosinophils, and platelets] contributing to IgE-mediated allergic pathophysiology (137). The “low affinity” FcεRII, first discovered on B cells, is also expressed on several other cell types, including various APCs, and also airway and gut epithelial cells (137). FcεRI-dependent MC activation occurs following exposure to allergens, bacterial and viral superantigens, and IgE antibodies. This pathway has been targeted successfully with omalizumab, which prevents IgE binding to the FcεRI and has been approved for use in asthma and chronic urticaria (138, 139). Indeed, anti-IgE therapy with omalizumab, added to medium- or high-dose inhaled glucocorticoids, has proven effective in the treatment of patients with moderate-to-severe and severe allergic (IgE-mediated) asthma by reducing exacerbations and associated use of systemic glucocorticoids in addition to improving other clinical outcomes (140, 141). Since omalizumab reduces the expression of FcεRI on circulating basophils and MCs, it seems to lower the activity potentials of basophils and MCs, thereby reducing the potential reactivity of these cells. Concordantly, serum tryptase was reported to decrease under omalizumab therapy in two mastocytosis patients, but it remained unchanged in two other patients (142). A recent study performed on 18 non-atopic asthmatic patients showed improved lung function and reduced total bronchial mucosal IgE^+^ cells in bronchial biopsies, but not changed total MCs, plasma cells, B lymphocytes, eosinophils, and plasmablast (143). A pooled analysis of five randomized, double-blind, placebo-controlled trials demonstrated that the reduction of serum-free IgE by omalizumab was associated with a reduction in peripheral eosinophil counts in patients with moderate-to-severe asthma receiving moderate-to-high doses of glucocorticoids [see Stokes in this issue and Ref. (144)]. Smaller studies already reported an inhibitory effect of omalizumab on eosinophils, in the peripheral blood and in the sputum or in bronchial biopsies, but no significant results on tissue MC numbers (145–149). A decrease in blood eosinophilia during omalizumab therapy was proposed as predictor of less asthma exacerbations (150) as well as higher IL-13 levels in sputum predicted the response to omalizumab (151). However, despite these clinical evidences, the mechanisms whereby reductions in circulating IgE lead to a reduction in eosinophils remain unclear. It is possible that omalizumab leads to the inhibition of the release of pro-inflammatory mediators, cytokines, and chemokines from MCs/basophils or inhibition of the allergen-induced differentiation of T cells to Th2 cells by reducing the expression of FcεRI on APCs. Indeed, omalizumab was shown to reduce IL-4^+^ cells in the bronchial submucosa (145). A reduction in circulating IL-13 has also been reported in patients with moderate-to-severe allergic asthma treated with omalizumab (147). A decrease in eotaxin expression in exhaled breath condensate, exhaled NO, eosinophil blood count, serum ECP after 16 weeks of omalizumab treatment was observed (152). Increased eosinophil apoptosis and reduced numbers of GM-CSF^+^ lymphocytes have been observed in peripheral blood of omalizumab-treated patients with coexisting allergic asthma and rhinitis, which may also contribute to the inhibitory action of omalizumab on eosinophils (146). A direct effect of omalizumab on eosinophils may be possible *via* the FcεRI that have been detected on eosinophils, even though their functional significance has yet to be established (153).

Given the pivotal role played by eosinophils in the pathogenesis of severe eosinophilic asthma (3, 154), targeting IL-5 or IL-5Rα appears an interesting therapeutic approach (3, 131). Several randomized, double-blind, placebo-controlled studies demonstrated that mepolizumab (155, 156), reslizumab (157, 158), and benralizumab (159, 160) improved lung function and decreased asthma exacerbations in adult patients with severe eosinophilic asthma.

### Eosinophilic Esophagitis

Eosinophils, normally present in the gastrointestinal tract, are absent in the esophagus of healthy subjects. Eosinophilic esophagitis (EoE) is a chronic, immune-mediated esophageal disease, characterized by dysphagia, abdominal pain, and presence of ≥15 eosinophils/field at 400× magnification in the proximal and distal esophagus (161). In EoE, eosinophils are present in all layers of the esophagus, but predominate in the lamina propria and submucosal regions, and are considered the main effector cells in this disorder (161). Activated MCs and their products (e.g., TGF-β) have been described in the esophageal biopsies of active EoE patients (162, 163). The relative contribution of MCs and eosinophils to disease pathogenesis is still under investigation. There is no evidence supporting MC-targeting therapies in EoE (164–166). However, an open label, single arm, unblinded small study showed a statistically significant reduction in MCs and eosinophils in endoscopic biopsies of EoE patients following omalizumab treatment, which correlated with clinical outcome (167). IL-5 targeting therapies resulted in a reduction of esophageal inflammation, but only in minimal symptom relief (168). Interestingly, mepolizumab did not deplete eosinophils nor MCs in the duodenal mucosa of patients (169). In contrast, a pediatric retrospective study showed a reduction in esophageal eosinophil numbers upon mepolizumab treatment, which was more pronounced in a subgroup of responders that also displayed a marked reduction of tryptase^+^ MCs after treatment. These esophageal MCs were found adjacent to eosinophils, and the frequency of these MC/eosinophil couplets in the esophagus of the responders was reduced after mepolizumab treatment. Moreover, activated MBP^+^ eosinophils and unidentified cells adjacent to tryptase^+^ MCs in the esophagus produced IL-9, a pleiotropic cytokine with a pivotal role in activation and maturation of MCs. Interestingly, the authors reported that the esophageal MC numbers correlated with the severity of EoE symptoms, but the reduction of eosinophil numbers did not correlate with symptoms severity. In the subgroup of patients with a greater than 70% decrease in MC density, numbers of MCs correlated with the severity of symptoms. By contrast, there was no correlation between eosinophil numbers and symptom severity. This study suggests an additional role for eosinophils in EoE, as providers of IL-9 that promotes esophageal mastocytosis and indicates that interactions between MCs and eosinophils can regulate the severity of EoE symptoms (170). Reslizumab reduced intraepithelial esophageal eosinophils without improvements in symptoms (171). Thus, although the involvement of eosinophils and presumably MCs in EoE is likely, their relative contribution to the pathogenesis and symptoms of EoE is not fully understood.

### Eosinophilic Granulomatosis with Polyangiitis (EGPA)

Eosinophilic granulomatosis with polyangiitis, previously known as Churg–Strauss syndrome, is characterized by increased blood level of IL-5 and eosinophilia in peripheral blood and affected tissues (172). In EGPA, eosinophilic inflammation affects the upper (chronic rhinosinusitis) and lower airways (asthma) (173). Endocardial inflammation, coronary vasculitis, and pericarditis can be observed in patients with EGPA (79, 80). A preliminary study in a small group of EGPA patients demonstrated the efficacy of mepolizumab in reducing blood eosinophils, but not in improving the pulmonary functions (174). A recent multicenter, double-blind, parallel-group, phase 3 trial demonstrated that in patients with EGPA mepolizumab (300 mg s.c. every 4 weeks) was associated with more accrued time in remission than was placebo, which allowed for reductions in the glucocorticoid dose over a period of 52 weeks (175). We have found that omalizumab resulted in clinical improvement of asthma, reduction of peripheral blood eosinophils, and prednisone administration in EGPA patients (173, 176). However, the role of MC in the pathogenesis of EGPA is not fully understood.

### Eosinophilic Endomyocarditis and Atherosclerosis

The association between endomyocardial disease and eosinophilia was first described by Löffler in 1936 (177). Cardiac involvement is the most common cause of morbidity and mortality in patients with hypereosinophilia (3, 81–84). Eosinophils and their granule proteins have been found in cardiac biopsies from patients with eosinophilic endomyocardial disease (178, 179). Recently, an association of EoE and cardiomyopathy has been reported (180).

Eosinophil cationic protein and, to a lesser extent, MBP stimulate the release of preformed (histamine and tryptase) and the *de novo* synthesis of PGD_2_ and LTC_4_ from human HHMC (8, 181). Activated HHMCs release histamine and CysLTs, which exert profound cardiovascular and metabolic effects (182, 183). In addition, MBP and eosinophil peroxidase induce platelet aggregation (184). These observations suggest that infiltrating eosinophils and their mediators contribute to cardiac dysfunction in patients with eosinophilia.

Activated MCs are increased at site of atheromatous rupture in myocardial infarction (10). MCs in human coronary plaques release angiogenic factors, such as FGF-β (11), which enhance atherosclerotic plaque progression. Cardiac MC-derived renin promotes local angiotensin formation leading to cardiac dysfunction (12). Activated MCs may also promote abdominal aortic aneurysms (13, 14) presumably through the release of chymase (185, 186) and CysLTs (15).

### Skin Disorders

Bullous pemphigoid (BP) is the most frequent autoimmune blistering dermatosis, characterized by autoantibodies directed against the dermal–epidermal junction proteins BP180/BP230 typically causing pruritic bullous eruptions. The immune response leading to blister formation in BP involves different inflammatory cells and molecules, including CD4 T cells, B cells, complement factors, neutrophils, as well as MCs and eosinophils (187). Serum levels of ECP were elevated in patients with active BP compared with healthy controls. Moreover, MC tryptase serum levels were associated with circulating anti-BP180 autoantibodies and decreased at the time of clinical remission (188). In a murine model of BP, blistering was dependent on C5a–C5aR interaction on MCs, which led to the activation of the p38 MAPK pathway in MC and their degranulation (189). Moreover, blood, skin, and blister-derived eosinophils were activated in patients with BP compared to controls. Activated eosinophils produced CCL26, IL-6, IL-8, and IL-1α in BP skin and blister fluid and displayed apoptosis features (190). Interestingly, IL-5-activated eosinophils were shown *ex vivo* to directly contribute to BP blister formation in the presence of BP autoantibodies. Indeed, IL-5-activated eosinophils induced dermal–epidermal separation, which was dependent on eosinophil adhesion, FcγR activation, ROS production, degranulation, and eosinophil extracellular trap formation (191).

Psoriasis is a frequent, chronic recurrent inflammatory skin disease which results from dysregulation between environmental factors, epithelial cells and immune cells (100). MCs infiltrate skin lesions of psoriatic patients and were identified as high producers of IL-17A and IL-22, both cytokines involved in psoriasis pathogenesis (192, 193). MCs and keratinocytes also induced angiogenesis by producing IL-8 and VEGF-A (194). In contrast, eosinophils were not increased in the skin or peripheral blood of psoriatic patients.

Atopic dermatitis (AD) is a common chronic inflammatory skin disease driven by specific genetic and immunological mechanisms (100). MC-derived histamine, tryptase, chymase, and other inflammatory mediators contribute to itching and inflammation in patients with AD (195). However, MCs were not required for the development of disease in a murine model of AD (196). AD is characterized by an increased number of circulating eosinophils and dermal and epidermal infiltrates of eosinophils. Tissue and blood eosinophilia and increased circulating levels of ECP, MBP, and eosinophil-derived neurotoxin have been correlated with disease activity. Serum levels of IL-5 were increased in AD patients and correlated with disease activity. However, although eosinophils might have important roles in AD pathogenesis, their exact mechanisms are not fully understood (197).

### Tumor-Associated MCs (TAMCs) and Tumor-Associated Eosinophils in Cancer

Tumor-associated eosinophilia was first described in 1893 (198). Eosinophilia is frequently observed in patients with solid tumors (199–201) and Hodgkin’s lymphoma (202). Eosinophils are recruited to tumors by chemoattractant CCL11 (eotaxin-1), which binds to CCR3 (203) and damage-associated molecular patterns, notably the alarmin high-mobility group box 1, released by necrotic tumor cells (204, 205).

Clinical studies addressing the role of eosinophils in tumors provided conflicting results. Tumor-associated eosinophilia was related to good prognosis in colorectal, head and neck, bladder and prostate cancers (206–208). By contrast, in Hodgkin’s lymphoma, oral squamous cell carcinoma, and cervical carcinoma, eosinophils have been linked to poor prognosis (206, 207, 209, 210).

Experimental studies also provided inconclusive results (211). Indeed, human eosinophils exert tumoricidal activity toward cancer cells through the release of TNF-α and granzyme A, contained in their secondary granules (17, 212). On the other hand, tumor-recruited eosinophils influence tumor angiogenesis, through distinct mechanisms. Human eosinophils and their supernatants induce endothelial cell proliferation *in vitro* and angiogenesis *in vivo* (213). Eosinophils contain VEGF in their secretory granules that can be secreted upon activation by IL-5 (127). In addition, eosinophils can contribute to tumor angiogenesis through the release of other proangiogenic molecules such as OPN (94) and MBP (129). Recently, activated eosinophils were shown to be essential for tumor rejection (16). Indeed, tumor-homing eosinophils secreted chemoattractants such as CCL5, CXCL9, and CXCL10, which recruited CD8^+^ T cells to the tumor (16).

Tumor-associated MCs are present in several human solid (41–55) and hematologic tumors (56–64). Peritumoral and/or intratumoral MC density is increased in different types of human cancer (18). Although the role of MCs and their mediators in experimental and human tumors is still controversial (19, 214), the bidirectional interaction between MCs and eosinophils can influence tumor angiogenesis and lymphangiogenesis.

Tumor immunologists have just scratched the surface of the complexity of the multidirectional interactions between eosinophils, MCs, and their neighboring tumor cells in tumor microenvironment.

### MCs and Eosinophils in Myeloproliferative Disorders

Mast cells and eosinophils can also found to be increased in primary myeloproliferative disorders of the bone marrow. The mechanism of the increased numbers of MCs and eosinophils in myeloproliferative disorders involves a primary defect in a tyrosine kinase gene resulting in uncontrolled proliferation and dysregulated apoptosis. Two such disorders are particularly associated with increased numbers of both cell types: systemic mastocytosis (SM) and chronic eosinophilic leukemia (CEL).

Mastocytosis is an abnormal clonal MC expansion and accumulation in several tissues including the bone marrow and the skin (215, 216). Cutaneous mastocytosis is associated with gain-of-function *Kit* mutations in approximately 8% of cases (217). Almost all patients with SM present a somatic mutation in codon 816 (D816V) of the gene encoding the receptor Kit, which leads to the substitution of a valine for an aspartate in the protein. Because of the D816V mutation, Kit is constitutively active, resulting in autophosphorylation and enhancement of MC differentiation and survival. A variable percentage (15–28%) of patients with SM also presents peripheral blood eosinophilia, which predicted poorer prognosis in some studies (218–220). In patients with cutaneous or SM, a correlation between the levels of soluble IL-5Rα (sIL-5Rα) and eosinophils in peripheral blood was also found (219).

In 2003, the FIP1L1–PDGFRA fusion was identified in patients with idiopathic hypereosinophilic syndrome and its presence redefined such patients having a neoplasm instead of idiopathic hypereosinophilic syndrome. Before the discovery of this cytogenetic rearrangement, the patients carried a poor prognosis due to early cardiac death in the absence of effective treatment. The identification of this fusion rearrangement as therapeutic target of imatinib dramatically changed the perspectives of these patients, due to a prompt hematologic and clinical remission. Patients with FIP1L1–PDGFRA^+^ CEL exhibit features of myeloproliferative syndromes such as splenomegaly, hypercellular bone marrow, and clinicopathological aspects that overlap with systemic MC diseases, such as increased number of abnormal MCs, elevated circulating tryptase levels, and bone marrow fibrosis (221). These similarities raised the doubt that FIP1L1–PDGFRA^+^ CEL could be considered a subtype of SM, rather than a primary eosinophil disease (222). Indeed, even though less dense clusters of MCs compared to the typical multifocal aggregates of D816V Kit^+^ SM, in some cases MCs exhibited spindle-shaped morphology and aberrant surface expression of CD25, both minor criteria for SM according to the WHO criteria (223). In the revised 2008 WHO semi-molecular classification of myeloid neoplasms, FIP1L1–PDGFRA^+^ disease is not considered a subtype of SM. To date, FIP1L1–PDGFRA and D816V *Kit* mutations appear to be mutually exclusive. In the D816V Kit^+^ patients, gastrointestinal symptoms, urticaria pigmentosa, thrombocytosis, median serum tryptase value, and the presence of MC dense infiltrates in the bone marrow were increased compared to patients with FIP1L1–PDGFRA mutation. By contrast, cardiac and pulmonary symptoms, median eosinophil count, eosinophil to tryptase ratio, and serum B_12_ levels were higher in the FIP1L1–PDGFRA^+^ patients. Whether a patient with peripheral eosinophilia and increased bone marrow MC infiltration carries a D816V Kit or FIP1L1–PDGFRA mutation is important for guiding therapeutic decisions. Indeed, FIP1L1–PDGFRA mutation is highly sensitive to imatinib treatment, which induces clinical remission as early as 4 weeks. By contrast, the vast majority of SM carrying D816V Kit mutation are imatinib resistant and candidate to second-line tyrosine kinase inhibitors or cytoreductive therapy (224).

## Conclusion

Mast cells and eosinophils were identified and named by Paul Ehrlich based on their capacity to be stained by specific dyes. These cells and their mediators have been classically associated with the pathogenesis of allergic disorders. However, there is now evidence that MCs and eosinophils are involved in autoimmune disorders, vasculitis, cardiovascular diseases, as well as solid and hematologic tumors. MCs and eosinophils play complex, sometimes complementary, but also distinct roles in these conditions. The latter findings are not surprising given the observations that these cells have distinct myeloid progenitors, are activated by different agonists, and differ morphologically, ultrastructurally, immunologically, biochemically, and pharmacologically.

In allergic disorders (e.g., asthma, allergic rhinitis, chronic urticaria) and certain solid (e.g., gastric and prostate cancers) and hematologic tumors (e.g., Hodgkin’s lymphoma), MCs and eosinophils can be found in close proximity. In particular, in allergic diseases, these cells can form the AEU (65). It is now clear that MCs modulate several eosinophil functions through the release of a plethora of preformed (e.g., SCF, histamine, and adenosine) and *de novo* synthesized mediators (e.g., IL-5, LTC_4_, SCF, PGD_2_, and PAF). On the other side, eosinophils modulate MC functions through the production of several mediators (e.g., IL-5, PAF, ECP, MBP, and NGF). These bidirectional interactions between MCs and eosinophils might be important not only in allergic diseases but also in several inflammatory and neoplastic disorders.

## Author Contributions

All authors listed have made a substantial, direct, and intellectual contribution to the work and have approved the final version of the manuscript.

## Conflict of Interest Statement

The authors declare that the research was conducted in the absence of any commercial or financial relationship that could be construed as a potential conflict of interest.

## Abbreviations

SCF, stem cell factor; MCs, mast cells; H_4_R, histamine 4 receptor; PAR-2, protease-activated receptor 2; ECP, eosinophil cationic protein; MBP; major basic protein; MRGPRX2, Mas-related G protein-coupled receptor member X2; OPN, osteopontin; IL-5, interleukin 5; GM-CSF, granulocyte-macrophage colony-stimulating factor; LTC_4_, leukotriene C_4_; PGD_2_, prostaglandin D_2_; PAF, platelet-activating factor; NGF, nerve growth factor; VEGF-A, vascular endothelial growth factor A; IL-5R, interleukin 5 receptor; CysLTR_1/2_, cysteinyl leukotriene receptor 1/2; CRTH2, chemoattractant receptor-homologous molecule expressed on Th2 cells; PAFR, platelet-activating factor receptor; TrkA, tyrosine kinase receptor A; VEGF-R1/2, vascular endothelial growth factor receptor 1/2.
